# Reappraising the Role of Trans-Sphenoidal Surgery in Prolactin-Secreting Pituitary Tumors

**DOI:** 10.3390/cancers13133252

**Published:** 2021-06-29

**Authors:** Pier Paolo Mattogno, Quintino Giorgio D’Alessandris, Sabrina Chiloiro, Antonio Bianchi, Antonella Giampietro, Alfredo Pontecorvi, Laura De Marinis, Alessandro Olivi, Carmelo Anile, Liverana Lauretti

**Affiliations:** 1 Institute of Neurosurgery, Fondazione Policlinico Gemelli IRCCS, Università Cattolica del Sacro Cuore-Roma, Largo A. Gemelli 8, 00168 Rome, Italy; giorgiodal@hotmail.it (Q.G.D.); alessandro.olivi@policlinicogemelli.it (A.O.); carmelo.anile@policlinicogemelli.it (C.A.); liverana.lauretti@unicatt.it (L.L.); 2 Pituitary Unit, Department of Endocrinology and Metabolism, Fondazione Policlinico Gemelli IRCCS-Università Cattolica del Sacro Cuore-Roma, Largo A. Gemelli 8, 00168 Rome, Italy; schiloiro@gmail.com (S.C.); antonio.bianchi@policlinicogemelli.it (A.B.); antonella.giampietro@policlinicogemelli.it (A.G.); alfredo.pontecorvi@policlinicogemelli.it (A.P.); laura.demarinis@policlinicogemelli.it (L.D.M.)

**Keywords:** prolactinoma, transsphenoidal surgery, dopamine agonists, DA withdrawal, cure rate

## Abstract

**Simple Summary:**

Prolactinomas constitute a subgroup of pituitary adenomas for which there are several treatment options. Dopamine agonists (DA), since their introduction, have shown a strong efficacy both in the control of hyperprolactinemia and of the significant volumetric reduction of prolactinomas, leading, in some cases, to a definitive cure. Trans-sphenoidal surgery (TSS) has been traditionally confined to a failure of medical therapy, pituitary apoplexy with neurological worsening, and prolactinomas with wide cystic components. Moreover, the recent technical innovations introduced in TSS and increasing experience of surgeons have allowed to achieve better results, such as complete tumor resection with lower complication rates. On these grounds, the authors reviewed the extensive institutional Prolactinomas case series over the last 25 years to analyze the role of TSS in the management of Prolactinomas, particularly in terms of the cure rate.

**Abstract:**

Background: Prolactinomas represent a unique challenge for endocrinologists and neurosurgeons. Considering recent innovations in surgical practice, the authors aimed to investigate the best management for prolactinomas. Methods: A retrospective, cross-sectional and monocentric study was designed. Consecutive patients affected by prolactinomas were enrolled if treated with a first-line treatment with a dopamine agonist (DA) or trans-sphenoidal surgery (TSS). Patients carried giant prolactinomas, and those with a follow-up <12 months were excluded. Results: Two hundred and fifty-nine patients were enrolled. The first treatment was DA for 140 patients and TS for 119 cases. One hundred and forty-six of 249 patients (58.6%) needed a second therapy. The mean follow-up was 102.2 months (12–438 months). Surgery highly impacted on the cure rate—in particular, in females (*p* = 0.0021) and in microprolactinomas (*p* = 0.0020). Considering the multivariate analysis, the female gender and surgical treatment in the course of the clinical history were the only independent positive predictors of a cure at the end of 5 years follow-up (*p* = 0.0016, *p* = 0.0005). The evaluation of serum prolactin (24 hours after TSS) revealed that 86.4% of patients with postoperative prolactin (PRL) ≤10 ng/mL were cured at the end of the follow-up (*p* < 0.0001). Conclusions: According to our experience, surgery allows a high cure rate of prolactinomas, particularly in females with microadenoma, with a good safety profile. TSS for prolactinomas should be considered as a concrete option, during the multidisciplinary evaluation, in centers of reference for pituitary diseases.

## 1. Introduction

Pituitary adenomas, or pituitary neuroendocrine tumors (WHO 2017), account for 10–15% of primary intracranial neoplasms. Among secreting pituitary tumors, prolactinomas are the most common (40–50% of the total) [1]. Prolactinomas, with their various biological and clinical features, might represent an oncological and neurosurgical challenge. Dopamine agonists (DA) are considered as a first-line treatment due to their efficacy and safety profile [2,3], while trans-sphenoidal surgery (TSS) has been confined to a failure of medical therapy, pituitary apoplexy with neurological worsening, and prolactinomas with wide cystic components. [4]. However, data on a definitive cure with DA are debated [2,4], and many patients remain on lifelong therapy to avoid disease relapse. On the other hand, several papers reported good results with TSS as the first-line therapy for prolactinomas [5,6,7,8]. Moreover, the recent technical innovations introduced in TSS, such as high-definition surgical endoscopes and extremely reliable neuronavigation systems, have made it possible to further expand the surgical possibilities and to obtain better results, such as complete tumor resection with lower complication rates. [9] Thus, in the neuro-oncological/endocrinological community, a debate on the possibility to expand the traditional indications of TSS as the first-line treatment for prolactinomas is alive [10,11].

In the present study, we conducted a thorough analysis of the surgical and medical experiences of over 25 years in the treatment of prolactinomas with the aim to verify the role of surgery. 

## 2. Materials and Methods

### 2.1. Study Design

We conducted a retrospective and cross-sectional study, reviewing the clinical, radiological, and surgical charts of consecutive patients enrolled according to the following inclusion criteria: (1) diagnosis of prolactinoma, (2) treatment with DA (cabergoline or bromocriptine) or/and with neurosurgical operation via TSS (either endonasal endoscopic or sublabial microsurgical), and (3) diagnosis and treatment of prolactinomas conducted at our institution between 1 January 1992 and 31 December 2016. 

Excluded from the study:

1. Patients carrying giant prolactinomas (diameter > 4 cm);
2. Patients with a follow-up shorter than 12 months.

All the patients provided informed consent, agreeing to the research principles of the Institutional Ethics Committee.

Two hundred and fifty-nine patients (164 women and 95 men) fulfilled the criteria for enrollment in the present study. The baseline characteristics of the patients are detailed in Table 1.

### 2.2. Clinical Management of Patients

For each patient, the diagnosis of prolactinoma was made following the international guidelines [4]. All patients diagnosed as having a prolactinoma were evaluated by a multidisciplinary team with a neuro-endocrinologist and a neurosurgeon. According to Klibanski [12], TSS was offered as the first-line treatment in the case of pituitary apoplexy, and macroprolactinomas in patients with a psychiatric disorder (for which dopamine agonists are contraindicated); contrarily to Klibanski, we recommended surgery as the first choice, even in macroadenomas determining visual field defects and in the case of microadenomas in fertile women with a pregnancy desire. Each time, the therapeutic strategy was jointly discussed between the endocrinologists, neurosurgeons, and the patient, considering the risks and benefits, with the final choice based upon the patient’s preference. 

### 2.3. Follow-Up

During the follow-up, all patients underwent: assessment of their prolactin levels one and three months after pituitary surgery/the start of DA therapy and then every six months and a pituitary MRI three and six months after pituitary surgery/the start of DA therapy and then every year. 

The outcome at the end of the follow-up was classified as: (1) cured in the cases of the regression of clinical symptoms, normalization of basal prolactin levels (below the gender-specific normal upper limit), and absence of neuroradiological evidence of a residual/recurrent tumor after at least 12 months from the neurosurgical treatment or DA discontinuation, (2) controlled disease in the cases with normal serum PRL concentrations and stable neuroimaging during DA therapy, and (3) uncontrolled disease in the cases with high serum PRL concentrations (above the gender-specific normal upper limit) and/or tumor progression on neuroimaging during DA therapy. Outcomes (2) and (3) were grouped into “not cured”.

### 2.4. Statistical Analysis 

Continuous variables were expressed as the mean (range), and categorical variables as the absolute and relative frequency. A comparison of the continuous variables between the groups was performed using the Mann–Whitney *U* test. A comparison of the categorical variables was performed by chi-square statistics using Fisher’s exact test when appropriate. A multivariate analysis model was built using logistic regression to calculate the odds ratio (OR) of a cure by adjusting for the following parameters: size of the adenoma, gender, surgical treatment in the course of clinical history, type of first treatment, and cavernous sinus invasion. *p* < 0.05 was considered significant. A ROC curve was built to assess the diagnostic accuracy for a cure of postoperative serum prolactin; the value with the highest Youden index (sensitivity-(1-specificity)) was designed as the best cut-off. StatView ver. 5.0 software was used (SAS Institute, Cary, NC, USA).

## 3. Results

### 3.1. Patient Population

As detailed in Table 1, 64% of female patients were affected by microadenomas, whereas the majority of male patients harbored a macroadenoma (78.9%; *p* < 0.0001, Fisher’s exact test). The mean age was 35.2 years; females were significantly younger than males (*p* < 0.0001, Mann–Whitney *U* test). The mean follow-up was 102.2 months (range, 12–438 months) and did not differ significantly between genders.

At the last follow-up, 113 patients (43.6% of cases) were considered cured. The remaining 146 patients (56.4%) were not cured, carrying either a controlled or an uncontrolled disease.

### 3.2. Impact of Surgery as the First-Line Treatment

Surgery as the first-line treatment was offered to 45.9% of the patients, and the detailed data are provided in Table 1. Overall, surgery as the first-line treatment did not significantly impact the rate of a cure (Table 2). Female patients harboring microadenoma buildup the subgroup of patients received the best advantage from surgery as their first-line therapy.

### 3.3. Impact of Surgery as the Second-Line Treatment

About half of the patients initially treated with DA received surgery as a second-line treatment due to an intolerance or resistance to medical therapy. Thus, overall, 194 out of 259 patients (74.9%) were surgically treated during the study period. Surgery determined a significant increase in the rate of a cure compared to DA alone in the whole cohort (50% vs. 24.6%, *p* = 0.0005, Fisher’s exact test). As detailed in Table 2, female patients, particularly those harboring microadenoma, still received the best advantage from surgery. Contrarily, surgery was not beneficial in males in terms of a cure rate.

### 3.4. Adjustment for Other Factors Impacting the Rate of a Cure

We then aimed at assessing the other parameters that had an impact on the final rate of a cure. The results are shown in Table 3. Female (*p* < 0.001), patients carrying microadenomas (*p* = 0.0057) and noncavernous sinus invasive adenomas (Knosp 0-II vs. III and IV, *p* = 0.02) had a higher cure rate at the end of the follow-up. 

In the multivariate analysis, surgery (either as the first- or second-line), female sex, and microadenoma emerged as independent prognosticators of a cure at the end of the follow-up (Table 3). Noteworthy, among these factors, surgery was the most significant (*p* < 0.0001) and had the highest odds ratio for a cure (5.1).

### 3.5. Validation of Results in Long-Term Follow-Up Patients

In order to validate the results on the positive role of surgery for a cure in prolactinomas, we explored the results in those patients with follow-ups of at least 5 years. 

One hundred and sixty-four patients met this criterion, 128 of which underwent TSS (as the first-line or as the second-line treatment). As shown in Table 4, TSS determined a significant increase in the cure rate both for the univariate (*p* = 0.0042) and the multivariate analyses (*p* = 0.0005), thus confirming our findings. 

### 3.6. Surgical Complications

No deaths occurred. The overall surgical complications rate was 3.6%. In detail: two patients (1.0%) had nasal aesthetic changes, four (2.1%) had CSF leaks needing reoperations, three (1.5%) needed chronic steroid replacement therapy, and two of them also had desmopressin. We assessed in detail the endocrinological outcomes in the subgroup of female patients harboring microprolactinomas aged less than 40 years and with a minimum follow-up of 5 years, (*n* = 37). In these patients, no postoperative deficits in the pituitary–adrenal axis nor in the gonadotropins axis were noticed; actually, the latter recovered back to normal.

### 3.7. Selection Biases

Due to the nonrandomized design of the study, surgery was not homogeneously distributed among the subgroups. As shown in Table 1, TSS was more frequent in males than in females (both as the first-line treatment, *p* > 0.0001, and during the disease course, *p* = 0.0045) and in macroadenomas compared to in microadenomas (*p* < 0.0001 both as the first- and second-line), whereas cavernous sinus invasion was homogeneously distributed among the groups.

### 3.8. First Day Postoperative Serum PRL as Biomarker for a Cure

**Figure 1:**
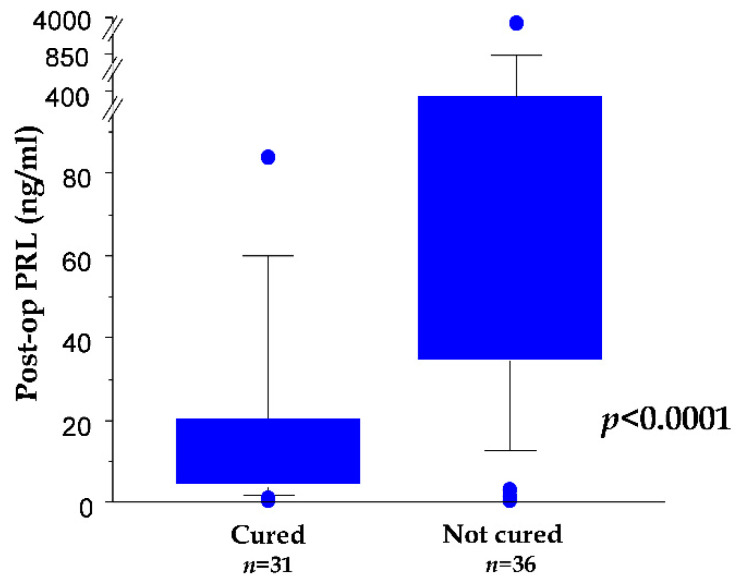
Box plot showing postoperation serum PRL in the subgroup of patients cured vs. not cured at the end of the 5-year minimum follow-up.

The dosage of PRL conducted in fasting conditions on the morning of the first day after surgery was significantly lower in cured vs. not cured patients (*p* < 0.0001, Mann–Whitney *U* test; Figure 1). The best cut-off was set at 37.5 ng/mL. Moreover, by applying a clinically relevant cut-off value of 10 ng/mL, 86.4% of patients with lower values vs. 27.3% of patients with higher values were cured at the end of the follow-up (*p* < 0.0001, Fisher’s exact test).

## 4. Discussion

In this study, we investigated the role of TSS in the treatment of prolactinomas, reviewing our cohort of patients that were managed with surgical and/or medical therapies over a period of 25 years. 

Our findings demonstrated that patients in which the adenoma was surgically removed had the highest probability of reaching a cure. In fact, in this cohort of patients, TSS acts as an independent positive prognostic factor for a cure for prolactinomas, together with female gender and microadenoma. Notably, in patients with a follow-up longer than 5 years, the only independent positive prognosticators for a cure remained surgery and female sex, thus confirming our assumptions. Moreover, the prognostic value of surgery was independent from the timing, thus highlighting its role when also performed as a second-line treatment after DA failure. 

Notably, our results allowed to identify a group of patients that may strongly benefit from the surgical removal of prolactinomas. In fact, we showed that female patients treated with first-line surgery had the highest rate of a cure, regardless of the tumor dimension; among them, women harboring microadenoma were the subgroup that benefited the most from surgery (Table 2). Importantly, in females of childbearing age harboring microprolactinomas, not only were there no postoperative pituitary adjunctive dysfunctions but, also, the previously altered gonadotropic axis recovered completely to normal in 70% of the cases.

According to the current guidelines [4], the first-line therapeutic choice for prolactinoma is medical therapy with DA, whereas surgery is usually confined to a complementary therapy for patients resistant to DA. However, recent studies have proved that a recurrence of prolactinoma may be observed in 20–77% of cases after the withdrawal of DA [13], matching with the tumoral dimension, invasion of the cavernous sinus, nadir prolactin value reached during DA treatment [14], persistence of the tumoral residual disease, and the duration of treatment with DA [15]. 

At our center, besides widely accepted indications (pituitary apoplexy, DA failure, and psychiatric disorders), surgery was also offered to patients harboring macroadenomas causing severe visual impairment or harboring “enclosed” prolactinomas deemed curable by surgery. This latter instance was particularly fitting for women of childbearing age, in whom obtaining a surgical cure avoided a lengthy medical treatment potentially interfering with pregnancy. Our evidence is in line with what has been published by high-volume centers [10,16,17,18,19,20,21,22,23,24,25,26,27]: TSS is linked to low surgical complications and a remission rate of about 80% and 40%, respectively, for micro- and macroprolactinomas. Consistently, Andereggen et al. [28] showed that, at 10 years follow-up, the control of hyperprolactinemia required DA agonist therapy in 32% of patients who underwent primary surgical therapy and in 64% of patients who received primary medical therapy and that primary surgical therapy is a protective factor for long-term treatment with DA. The low surgical complication rates reported in the literature were confirmed in our cohort.

It has also been suggested that the surgical removal of an adenoma should be part of a patients’ counseling for the decision of the initial treatment due to the low morbidity of this procedure if performed by an experienced neurosurgeon [29]. In addition, the availability of tumoral tissue may allow a detailed pathological analysis for investigating both the biomarkers of aggressiveness, such as Ki67, p53, the mitotic count, minichromosome maintenance 7 (MCM7), and estrogen receptors, and biomarkers of the treatment response, such as dopamine and somatostatin receptors [30,31,32,33]. In fact, a very detailed pathological analysis may facilitate the identification of cases with a high risk of recurrence and may orient towards a personalized therapy—in particular, in cases of difficult and aggressive prolactinomas [34].

The main limitations of our study were its retrospective design and the lack of randomization. The main selection biases were the prevalence of males and macroadenomas in surgical patients, probably due to visual disturbances as a reason for surgery. However, this study described the real-life scenario of a large and monocentric series of patients managed at a historical pituitary unit with a long-standing commitment toward research on prolactin in physiological and pathological conditions [35,36,37,38,39,40,41,42,43]. Moreover, the multivariate analysis was expected to reduce these biases. Another limitation was that, in this study, the complex endocrine syndrome induced by prolactinomas was not analyzed; focusing on a few selected serological and neuroradiological aspects of the illness was essential in order to reliably analyze a considerable number of patients with long-term follow-ups.

Among the strong points of the study was that, though collected over a 25-year timeframe, our monocentric series was highly homogeneous as concerns the indications, treatment, and follow-ups. To further reduce the confounding factors of our analysis, giant prolactinomas were excluded, because, in such invasive cases, a multimodality treatment, including radiotherapy and lifelong DA, is often required. 

## 5. Conclusions

TSS may represent a valid alternative to DA therapy, particularly in females with microadenomas, as it provides the highest chance of a cure at long-term follow-up. 

Concurringly with the aforementioned reports, our experience prompts surgery among the first-line possible treatments for the management of prolactinomas in reference centers.

## Figures and Tables

**Table 1:** Baseline characteristics of the study patients and first-line treatments.

Group	Whole Cohort	First-Line Treatment		Surgery	
		TSS	DA	Yes	No
*n* (%)	259 (100)	119 (45.9)	140 (54.1)	194 (74.9)	65 (25.1)
Female, *n* (%)	164 (63.3)	56 (34.1)	108 (65.9)	113 (68.9)	51 (31.1)
Male, *n* (%)	95 (36.7)	63 (66.3)	32 (33.7)	81 (85.3)	14 (14.7)
Microadenomas, *n* (%)	125 (48.3)	41 (32.8)	84 (67.2)	78 (62.4)	47 (37.6)
Macroadenomas, *n* (%)	134 (51.7)	78 (58.2)	56 (41.8)	116 (86.6)	18 (13.4)
Female: microadenomas, *n* (%)	105 (64.0)	29 (27.6)	76 (72.4)	62 (59.0)	43 (41.0)
Female: macroadenomas, *n* (%)	59 (36.0)	27 (45.8)	32 (54.2)	51 (86.4)	8 (13.6)
Male: microadenomas, *n* (%)	20 (21.1)	12 (60)	8 (40)	16 (80)	4 (20)
Male: macroadenomas, *n* (%)	75 (78.9)	51 (68)	24 (32)	65 (86.7)	10 (13.3)
Age, mean (range) (years)	35.2 (18–78)	35.6 (18–78)	34.8 (18–76)	33.9 (18–78)	38.9 (18–76)
Knosp: 0–II, *n* (%)	227 (87.3)	99 (83.2)	127 (90.7)	168 (74.3)	58 (25.7)
Knosp: III and IV, *n* (%)	33 (12.7)	20 (16.8)	13 (9.3)	26 (78.8)	7 (21.2)
Follow-up, mean (range) (months)	102.2 (12–438)	100.9 (12–438)	103.2 (12–420)	108.8 (12–438)	82.7 (12–300)

DA, dopamine agonist; TSS, trans-sphenoidal surgery.

**Table 2:** Rate of a cure depending on the first-line treatment and surgery in the whole cohort.

	Rate of Cure					
Group	First-Line Treatment		p *	Surgery		p *
	TSS, *n* (%)	DA, *n* (%)		yes	no	
Whole Cohort	56/119 (47.1)	57/140 (40.7)	0.3172	97/194 (50.0)	16/65 (24.6)	0.0005
Female	38/56 (67.9)	49/108 (45.4)	0.0081	72/113 (63.7)	15/51 (29.4)	<0.0001
Male	18/63 (28.6)	8/32 (25)	0.8102	25/81 (30.9)	1/14 (7.1)	0.1026
Microadenomas	25/41 (61.0)	41/84 (48.8)	0.2530	52/78 (78.8)	14/47 (21.2)	<0.0001
Macroadenomas	31/78 (39.7)	16/56 (28.6)	0.2028	45/116 (38.8)	2/18 (11.1)	0.0031
Female: microadenomas	20/29 (69.0)	37/76 (48.7)	0.0080	44/62 (71.0)	13/43 (30.2)	<0.0001
Female: macroadenomas	18/27 (66.7)	12/32 (37.5)	0.0370	28/51 (54.9)	2/8 (25)	0.1455
Male: microadenomas	5/12 (41.7)	4/8 (50)	>0.9999	8/16 (50)	1/4 (25)	0.5913
Male: macroadenomas	13/51 (25.5)	4/24 (16.7)	0.5566	17/65 (26.2)	0/10 (0)	0.1043

DA, dopamine-agonist; TSS, trans-sphenoidal surgery; *, Fisher’s exact test; *n*, number of patients.

**Table 3:** Rate of a cure depending on the first-line treatment and surgery in the whole cohort.

	Rate of Cure					
Group	First-Line Treatment		p *	Surgery		p *
	TSS, *n* (%)	DA, *n* (%)		yes	no	
Whole Cohort	56/119 (47.1)	57/140 (40.7)	0.3172	97/194 (50.0)	16/65 (24.6)	0.0005
Female	38/56 (67.9)	49/108 (45.4)	0.0081	72/113 (63.7)	15/51 (29.4)	<0.0001
Male	18/63 (28.6)	8/32 (25)	0.8102	25/81 (30.9)	1/14 (7.1)	0.1026
Microadenomas	25/41 (61.0)	41/84 (48.8)	0.2530	52/78 (78.8)	14/47 (21.2)	<0.0001
Macroadenomas	31/78 (39.7)	16/56 (28.6)	0.2028	45/116 (38.8)	2/18 (11.1)	0.00313
Female: microadenomas	20/29 (69.0)	37/76 (48.7)	0.0080	44/62 (71.0)	13/43 (30.2)	<0.0001
Female: macroadenomas	18/27 (66.7)	12/32 (37.5)	0.0370	28/51 (54.9)	2/8 (25)	0.1455
Male: microadenomas	5/12 (41.7)	4/8 (50)	>0.9999	8/16 (50)	1/4 (25)	0.5913
Male: macroadenomas	13/51 (25.5)	4/24 (16.7)	0.5566	17/65 (26.2)	0/10 (0)	0.1043

DA, dopamine-agonist; TSS, trans-sphenoidal surgery; *, Fisher’s exact test; *n*, number of patients.

**Table 4:** Rate of a cure depending on the first-line treatment and surgery in the whole cohort; considering the whole court of patients, surgery (*p* < 0.0001), female sex (*p* < 0.0009), and size (*p* < 0.0182) strongly impacted the cure rate; surgery (*p* < 0.0005) and female sex (*p* < 0.0016) were confirmed significative for the subgroup with a follow-up >5 years.

	Whole Court				>5ys FU population			
	*p*-Value	Odds Ratio	95% Lower	95% Upper	*p*-Value	Odds Ratio	95% Lower	95% Upper
Not cured: constant	0.5143	0.751	0.318	1.776	0.5060	0.695	0.237	2.033
Surgery: Yes	**<0.0001**	0.196	0.087	0.439	**0.0005**	0.158	0.056	0.445
Age	0.921	1.001	0.981	1.022	0.5640	1.007	0.982	1.033
Sex: Female	**0.0009**	0.323	0.166	0.627	**0.0016**	0.257	0.11	0.598
Size: Micro-PRL-omas	**0.0182**	0.469	0.250	0.879	0.0997	0.516	0.235	1.134
I treatment: TSS	0.6573	0.863	0.451	1.650	0.7586	1.131	0.516	2.475
Knosp III and IV	0.618	1.280	0.485	3.381	0.6406	0.758	0.237	2.427

Ys, years; FU, follow-up; TSS, trans-sphenoidal surgery; PRL, prolactin.

## Data Availability

The source data are available from the corresponding author upon reasonable request.
